# Impact of acute kidney injury on in-hospital outcomes in Chinese patients with community acquired pneumonia

**DOI:** 10.1186/s12890-021-01511-9

**Published:** 2021-05-01

**Authors:** Dawei Chen, Hongbo Yuan, Changchun Cao, Zhihe Liu, Linglin Jiang, Yan Tan, Ji Ding, Mengqing Ma, Wenjuan Huang, Xin Wan

**Affiliations:** 1 Department of Nephrology, Nanjing First Hospital, Nanjing Medical University, 68 Changle Road, Nanjing, 210006 Jiangsu China; 2 Department of Nephrology, Sir Run Run Hospital, Nanjing Medical University, Nanjing, Jiangsu China; 3 Department of Respiratory Medicine, Nanjing First Hospital, Nanjing Medical University, Nanjing, Jiangsu China; 4 Department of Cardiothoracic Surgery, Yizheng People’s Hospital, Yangzhou, Jiangsu China

**Keywords:** Acute kidney injury, Community acquired pneumonia, Outcomes, Chinese

## Abstract

**Background:**

Acute kidney injury (AKI) is a frequent complication of community acquired pneumonia (CAP). However, the impact of AKI on in-hospital outcomes of patients with CAP in the Chinese population remains unclear.

**Methods:**

Patients diagnosed with CAP were evaluated in this retrospective observational study. Multiple Cox regression models were employed to identify the association between AKI and in-hospital mortality and 30-day mortality, respectively.

**Results:**

A total of 4213 patients were recruited; 950 (22.5%) patients were diagnosed with AKI. Independent risk factors for AKI were age, male gender, hypertension, cardiac dysfunction, diabetes, chronic kidney disease, acute respiratory failure, use of diuretics, use of vasoactive drugs, and CURB-65. Cox proportional hazards regression revealed AKI, use of angiotensin receptor blocker, hypertension, CURB-65, acute respiratory failure, and use of vasoactive drugs to be independent risk factors for both in-hospital and 30-day mortality. Compared to patients without AKI, those suffering AKI were found to have 1.31-fold (HR 1.31, 95% CI, 1.04–1.66; *P* = 0.023) and 1.29-fold (HR 1.29, 95% CI, 1.02–1.62; *P* = 0.033) increased in-hospital and 30-day mortality risks, respectively. In addition, patients with AKI were likely to require admission to intensive care unit (ICU) (42.9% versus 11.4%; *P* < 0.001), mechanical ventilation (33.8% versus 9.3%; *P* < 0.001), invasive mechanical ventilation (25.9% versus 5.8%; *P* < 0.001), non-invasive mechanical ventilation (25.4% versus 7.1%; *P* < 0.001), and experienced a longer duration of hospital stay (14 days versus 10 days; *P* < 0.001) than those without AKI. However, no significant difference in ICU stay (11 days versus 10 days; *P* = 0.099) and duration of mechanical ventilation (8 days versus 8 days; *P* = 0.369) between AKI and non-AKI groups was found.

**Conclusion:**

AKI was common in Chinese patients with CAP. Patients with CAP who developed AKI had worse in-hospital outcomes.

**Supplementary Information:**

The online version contains supplementary material available at 10.1186/s12890-021-01511-9.

## Background

Community acquired pneumonia (CAP) is a leading cause of infectious death globally [1, 2]. The number of patients hospitalized with CAP in the USA is estimated to have increased to 1 million in 2020, with similarly large increases globally [3, 4]. A previous survey reported the 2-week prevalence of pneumonia to have reached approximately 11/1000 in China [5].

Acute kidney injury (AKI) is a frequent complication of CAP; the incidence of AKI in CAP patients was reported to range from 18 to 34% [6–8]. Chawla et al. previously reported patients with AKI coexisted with pneumonia were worse than patients with either pneumonia or AKI alone [9]. Even among patients diagnosed with non-severe pneumonia, AKI was associated with long-term mortality [7]. However, the impact of AKI on in-hospital outcomes of CAP patients in the Chinese population remains unclear.

## Methods

### Study participants

This study was approved by the Nanjing First Hospital Institutional Review Board. Due to the retrospective nature of its design, patient consent was waived. All patients evaluated were initially admitted to Nanjing First Hospital (Nanjing, China) from January 2014 to May 2017 and diagnosed with CAP. The inclusion criterion was patient discharged with a primary diagnosis of CAP. Exclusion criteria were: (1) patients with less than two repeated serum creatinine (SCr) values; (2) patients with a history remarkable for pre-existing end-stage renal disease requiring dialysis; and (3) patients lacking complete medical records. Finally, 4213 patients were included for analysis in this study.

### Definitions of CAP and AKI

Pneumonia diagnosed based on detection of interstitial infiltrate changes on chest CT or radiography in patients with one or more of: [10, 11] (a) core body temperature > 38.0 °C; (b) recent presence of cough, sputum, or dyspnea; or (c) peripheral white blood cell counts < 4 × 10^9^/L or > 10 × 10^9^/L. In addition, illness onset was specifically in the community, rather than in the health-care setting.

The definition of AKI in our study adhered to the Kidney Disease Improving Global Outcomes (KDIGO) criteria, which defined AKI as an increase in SCr levels by ≥ 1.5-fold from baseline within 7 days of illness onset or an increase in SCr levels by ≥ 0.3 mg/dL (26.4 μmol/L) within 48 h of illness onset [12]. According to KDIGO criteria, AKI was classified into three stages: stage 1 (defined by an increase in SCr by at least 0.3 mg/ dL or a 1.5 to 1.9 fold increase from baseline); stage 2 (an increase in SCr by 2.0–2.9 fold from baseline); and stage 3 (an increase in SCr by ≥ 3.0 fold from baseline, by ≥ 4.0 mg/dL or by initiation of dialysis) [12]. Baseline SCr values were defined as the lowest levels measured during hospitalization. Due to the lack of data concerning urine output, urine output standards were not considered in this study.

### Variables

Demographic and clinical data included sex, age, and history positive or negative for chronic obstructive pulmonary disease (COPD), chronic cor pulmonale, pulmonary arterial hypertension, hypertension, atrial fibrillation, cardiac dysfunction, diabetes, chronic kidney disease, cerebrovascular disease, cancer, rheumatic diseases, acute respiratory failure, statin use, angiotensin receptor blocker (ARB) use, diuretic use, and vasoactive drugs use, as well as confusion, uremia, respiratory rate, blood pressure, and age 65 years or older (CURB-65) score [13].

### In-hospital outcomes

The primary outcome was in-hospital mortality, while secondary outcomes were 30-day mortality, necessitation for intensive care unit (ICU) admission, employment of invasive, or non-invasive mechanical ventilation, the duration of ICU stay, the duration employment of mechanical ventilation, and the duration of hospitalization.

### Statistical analysis

Analyses were carried out using SPSS v22.0 (IBM Corporation, New York, USA). For non-normally distributed data, continuous variables were presented as medians and interquartile ranges. For normally distributed data, continuous variables were expressed as mean ± standard deviation (SD). All categorical variables were presented as frequencies. Fisher exact or chi-square tests were performed to compare categorical variables, while Mann–Whitney U or Student’s t tests were used to compare continuous variables. Univariate logistic regression analysis was used to identify potential risk factors for AKI. Furthermore, variables with *P* values < 0.05 in univariable analysis were further analyzed via multivariable logistic regression to identify independent risk factors for AKI. Discrimination was determined by the area under curve (AUC) with a 95% confidence interval (CI); calibration was evaluated via the Hosmer–Lemeshow goodness-of-fit test. In addition, univariate and multivariable Cox proportional hazards regression models were used to evaluate associations between AKI and in-hospital and 30-day mortality, respectively. The proportional hazards assumption was confirmed graphically; *P* values less than 0.05 were considered statistically significant.

## Results

### AKI incidence

In total, 4213 patients with CAP were recruited. Mean patient age was 70.7 ± 16.7 years and 60.7% of patients was male; 950 patients (22.5%) were diagnosed with AKI, of which 604 patients (14.3%) were classified as stage 1, 160 patients (3.8%) were classified as stage 2, and 186 patients (4.4%) were classified as stage 3.

### AKI characteristics

Patient demographic data, comorbidities, complications, medication use and illness severity score were shown in Table 1. Male gender (68.9% versus 58.3%; *P* < 0.001) and age (82 years versus 72 years; *P* < 0.001) had significant difference between the AKI group and the non-AKI group. No statistically significant comorbidities in chronic cor pulmonale, pulmonary arterial hypertension, cancer, and rheumatic diseases between non-AKI and AKI groups. However, COPD, hypertension, atrial fibrillation, cardiac dysfunction, diabetes, chronic kidney disease, and cerebrovascular disease were more common in the AKI group. Patients in the AKI group were more commonly complicated with acute respiratory failure (35.5% versus 11.3%; *P* < 0.001), and were more likely to have used statin, ARB, diuretic, and vasoactive drugs. In addition, patients with AKI also had a higher CURB-65 scores (2 versus 1; *P* < 0.001).

**Table 1:** Patient demographic, comorbidity, complication, medication use, and severity scoring in patients with and without acute kidney injury

Variable	Total (n = 4213)	AKI (n = 950)	Non-AKI (n = 3263)	*P* value
*Demographic*				
Age (years)	75 (62–83)	82 (74–87)	72 (59–82)	< 0.001
Men (%)	2557 (60.7)	655 (68.9)	1902 (58.3)	< 0.001
*Comorbidity, n (%)*				
COPD	479 (11.4)	129 (13.6)	350 (10.7)	0.015
Chronic cor pulmonale	148 (3.5)	33 (3.5)	115 (3.5)	0.940
Pulmonary arterial hypertension	128 (3.0)	37 (3.9)	91 (2.8)	0.081
Hypertension	2091 (49.6)	602 (63.4)	1489 (45.6)	< 0.001
Atrial fibrillation	485 (11.5)	180 (18.9)	305 (9.3)	< 0.001
Cardiac dysfunction	930 (22.1)	372 (39.2)	558 (17.1)	< 0.001
Diabetes	829 (19.7)	261 (27.5)	568 (17.4)	< 0.001
Chronic kidney disease	280 (6.6)	167 (17.6)	113 (3.5)	< 0.001
Cerebrovascular disease	1333 (31.6)	421 (44.3)	912 (27.9)	< 0.001
Cancer	376 (8.9)	93 (9.8)	283 (8.7)	0.288
Rheumatic diseases	117 (2.8)	29 (3.1)	88 (2.7)	0.557
*Complication, n (%)*				
Acute respiratory failure	705 (16.7)	337 (35.5)	368 (11.3)	< 0.001
*Medication Use, n (%)*				
Statin	777 (18.4)	234 (24.6)	543 (16.6)	< 0.001
ARB	585 (13.9)	161 (16.9)	424 (13.0)	0.002
Diuretic	1671 (39.7)	711 (74.8)	960 (29.4)	< 0.001
Vasoactive drugs	527 (12.5)	306 (32.2)	221 (6.8)	< 0.001
*Severity scoring*				
CURB-65	1 (1–2)	2 (2–3)	1 (0–1)	< 0.001

Abbreviation: AKI: acute kidney injury; COPD: chronic obstructive pulmonary disease; ARB: angiotensin receptor blocker

### Risk factors for AKI

Univariate analysis of potential risk factors between non-AKI and AKI patients was shown in Table 1. Independent risk factors for AKI were identified by multivariate logistic regression model. Independent risk factors for AKI included age (OR 1.01, 95% CI: 1.004–1.02, *P* = 0.004), male gender (OR 1.37, 95% CI: 1.14–1.65; *P* = 0.001), hypertension (OR 1.25, 95% CI: 1.03–1.51; *P* = 0.026), cardiac dysfunction (OR 1.26, 95% CI: 1.02–1.54; *P* = 0.031), diabetes (OR 1.26, 95% CI: 1.02–1.54; *P* = 0.031), chronic kidney disease (OR 3.70, 95% CI 2.76–4.96; *P* < 0.001), acute respiratory failure (OR 1.50, 95% CI 1.20–1.87; *P* < 0.001), use of diuretics (OR 2.78, 95% CI 2.27–3.41; *P* < 0.001), use of vasoactive drugs (OR 2.66, 95% CI 2.10–3.37; *P* < 0.001), and CURB-65 (OR 1.90, 95% CI 1.70–2.12; *P* < 0.001) (Table 2). The receiver operating characteristic curve (ROC) corresponded with an area under curve (AUC) of 0.828 (95% CI = 0.814–0.843). Calibration was assessed by the Hosmer–Lemeshow test, which was not significant (*P* = 0.361).

**Table 2:** Risk factors for acute kidney injury in patients with community acquired pneumonia

Variable	OR	95% CI	*P* value
Age	1.01	1.004–1.02	0.004
Men	1.37	1.14–1.65	0.001
Hypertension	1.25	1.03–1.51	0.026
Cardiac dysfunction	1.26	1.02–1.54	0.031
Diabetes	1.26	1.02–1.54	0.031
Chronic kidney disease	3.70	2.76–4.96	< 0.001
Acute respiratory failure	1.50	1.20–1.87	< 0.001
Diuretic	2.78	2.27–3.41	< 0.001
Vasoactive drugs	2.66	2.10–3.37	< 0.001
CURB-65	1.90	1.70–2.12	< 0.001

### Impact of AKI on in-hospital outcomes

**Fig. 1:**
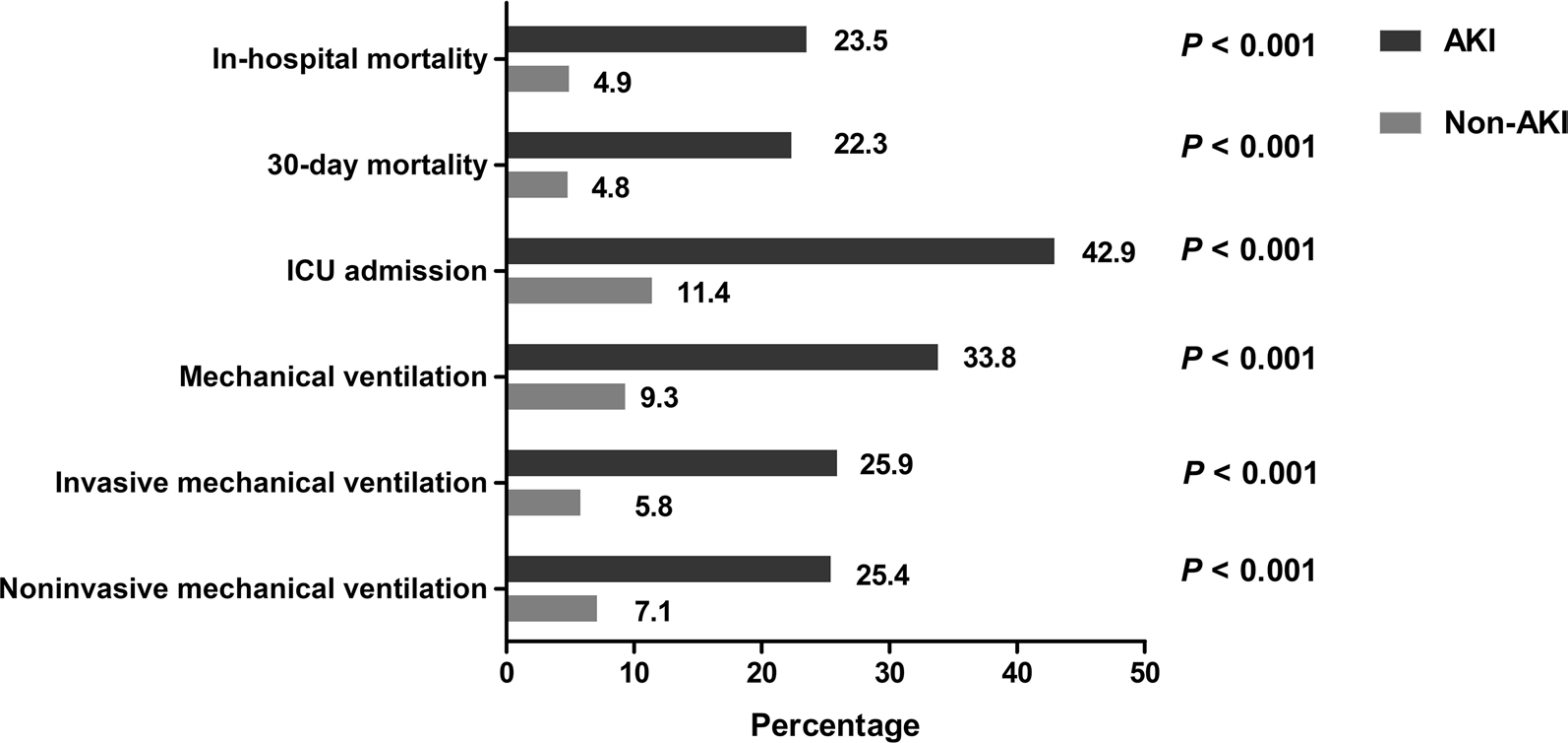
In-hospital outcomes for AKI versus non-AKI patients. *Notes*: This figure shows a comparison of in-hospital mortality, 30-day mortality, ICU admission, mechanical ventilation, invasive mechanical ventilation, and non-invasive mechanical ventilation between AKI and the non-AKI patients. The numbers at the top of the individual bars reflect the percentages of each outcome within this group. *P*-values were calculated using Pearson chi-squared/Fisher’s exact tests where appropriate. Abbreviations: AKI, acute kidney injury; ICU, intensive care unit

Overall, total in-hospital mortality was 9.1% (384/4213). In-hospital mortality rate was higher in patients with AKI than the non-AKI group (23.5% versus 4.9%%; *P* < 0.001) (Fig. 1) . After multivariable Cox proportional hazards regression, independent risk factors of in-hospital mortality were AKI (HR 1.31, 95% CI, 1.04–1.66; *P* = 0.023), use of ARB (HR 0.47, 95% CI, 0.33–0.67; *P* < 0.001), hypertension (HR 1.25, 95% CI, 1.01–1.55; *P* = 0.049), CURB-65 (HR 1.39, 95% CI, 1.25–1.55; *P* < 0.001), acute respiratory failure (HR 1.98, 95% CI, 1.52–2.58; *P* < 0.001), and use of vasoactive drugs (HR 2.76, 95% CI, 2.14–3.66; *P* < 0.001) (Additional file 1: Table S1). In comparison with patients with the non-AKI group, 30-day mortality was also higher in patients with AKI (22.3% versus 4.8%; *P* < 0.001) (Fig. 1). Independent risk factors of 30-day mortality were similar to those of in-hospital mortality (Additional file 1: Table S2).

Patients with AKI were more likely to require ICU admission (42.9% versus 11.4%; *P* < 0.001), mechanical ventilation (33.8% versus 9.3%; *P* < 0.001), invasive mechanical ventilation (25.9% versus 5.8%; *P* < 0.001), and non-invasive mechanical ventilation (25.4% versus 7.1%; *P* < 0.001) (Fig. 1). Patients with AKI had a longer length of hospital stay (14 days versus 10 days; *P* < 0.001) than those without AKI. Nevertheless, there was no significant difference in the length of ICU stay (11 days versus 10 days; *P* = 0.099) and length of mechanical ventilation (8 days versus 8 days; *P* = 0.369) between AKI and non-AKI groups.

### Impact of AKI stages on in-hospital outcomes

**Fig. 2:**
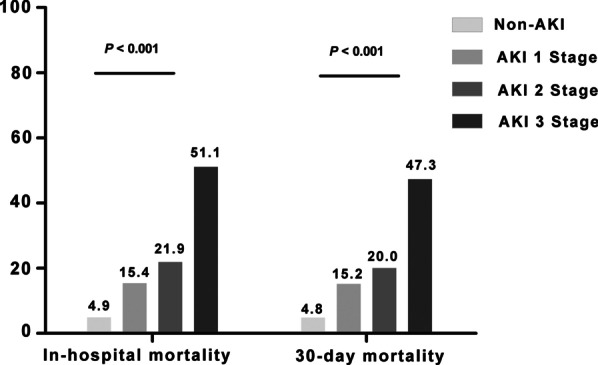
Impact of AKI stages on in-hospital and 30-day mortality. *Notes*: This figure details the percentages of in-hospital and 30-day mortality among patients suffering various stages of AKI. *P* < 0.001 for comparisons of primary outcomes among AKI stages 1, 2, and 3 and non-AKI patients (Pearson chi-squared test), respectively. Abbreviations: AKI, acute kidney injury

The in-hospital mortality rates of AKI stage 1, stage 2, and stage 3 were approximately 3.1-fold (15.4% versus 4.9%), 4.5-fold (21.9% versus 4.9%), and 10.4-fold (51.1% versus 4.9%) higher than that of non-AKI, respectively. Similarly, 30-day mortality rates of AKI stage 1, stage 2, and stage 3 were approximately 3.2-fold (15.2% versus 4.8%), 4.2-fold (20.0% versus 4.8%), and 9.9-fold (47.3% versus 4.8%) higher than that of non-AKI, respectively. In particular, 47.3% of patients with AKI stage 3 died with 30 days, and 51.1% of AKI stage 3 died during hospitalization (Fig. 2).

**Fig. 3:**
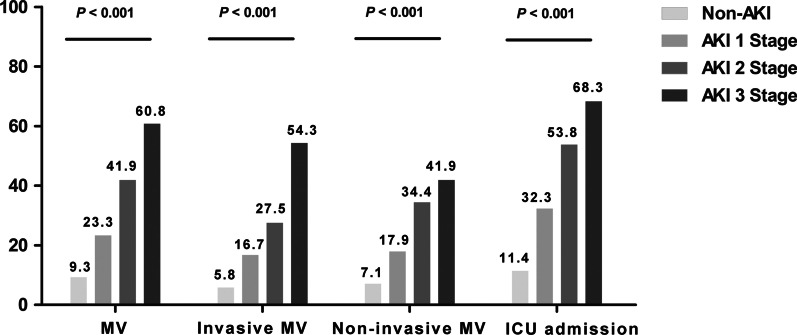
Impact of AKI stages on secondary outcomes. *Notes*: This figure details the percentages of secondary outcomes (ICU admission, mechanical ventilation, invasive mechanical ventilation, and non-invasive mechanical ventilation) among patients suffering every stage of AKI. *P* < 0.001 for comparisons of secondary outcomes among AKI stages 1, 2, and 3 and non-AKI patients (Pearson chi-squared test), respectively. Abbreviations: AKI, acute kidney injury; ICU, intensive care unit

Overall, the requirement of mechanical ventilation rates of AKI stage 1, stage 2, and stage 3 were approximately 2.5-fold (23.3% versus 9.3%), 4.5-fold (41.9% versus 9.3%), and 6.5-fold (60.8% versus 9.3%) higher than that of non-AKI, respectively. In this study, we found that patients with AKI stage 1 (17.9% versus 16.7%) and stage 2 (34.4% versus 27.5%) were more commonly requiring non-invasive mechanical ventilation than invasive mechanical ventilation. On the contrary, patients with AKI stage 3 (54.3% versus 41.9%) were more commonly requiring invasive mechanical ventilation than non-invasive mechanical ventilation. The ICU admission rates of AKI stage 1, stage 2, and stage 3 were approximately 2.8-fold (32.3% versus 11.4%), 4.7-fold (53.8% versus 11.4%), and 6.0-fold (68.3% versus 11.4%) higher than that of non-AKI, respectively. In particular, approximately half of AKI stage 2 and two-thirds of AKI stage 3 required intensive care. (Fig. 3).

## Discussion

To the best of our knowledge, this study may be the first study to explore the impact of AKI on in-hospital outcomes in Chinese patients with CAP. Our findings confirmed that patients suffering AKI experienced significantly worse in-hospital outcomes.

In our study, the incidence rate of AKI was nearly 22.5% in patients with CAP requiring hospitalization, similar to prior studies, which reported incidences ranging from 18 to 34% [6–8]. Moreover, we found that male gender, advanced age, hypertension, cardiac dysfunction, diabetes, chronic kidney disease, acute respiratory failure, use of diuretics, use of vasoactive drugs, and higher CURB-65 scores were independent risk factors for AKI in Chinese patients with CAP. Previous studies had reported that male gender, advanced age, hypertension, cardiac dysfunction, diabetes, chronic kidney disease, diuretic, and vasoactive drugs were traditional risk factors for AKI [14]. Acute respiratory failure was one common relevant CAP complication [15]. Acute respiratory failure was also reported to be a risk factor for AKI, with the incidence of AKI in patients suffering this condition ranging from 24 to 57% [15–17]. The kidney is particularly sensitive to minimal changes in oxygen, especially in the loops of Henle and proximal tubes. Severe hypoxemia was demonstrated to reduce renal blood flow [18, 19]. In addition, in this study we observed the CURB-65 was also a significant risk factor for AKI in Chinese patients with CAP. As such, patients with particular characteristics may benefit from further testing of SCr levels to detect AKI onset, as well as avoidance of nephrotoxic drugs use.

In this study, we found that patients suffering AKI experienced worse in-hospital outcomes. Patients had 4.8-fold risk of in-hospital mortality and 4.6-fold 30-day mortality higher than those with non-AKI. After multivariable Cox proportional hazards regression, patients developed AKI had 1.31 and 1.29-fold increased risks for in-patient and 30-day mortality, respectively. Ahsan et al. demonstrated that AKI was significantly associated with 30-day mortality (OR, 1.46; 95% CI; 1.04–2.04) [6]. Murugan et al. reported that patients with CAP who developed AKI suffered a 1.29-fold increased risk of death (HR, 1.29; 95% CI; 1.03–1.60) [7]. Lakhmir et al. reported that patients with pneumonia coexistent with AKI had significantly shorter time to death (HR, 1.17; 95% CI, 1.13–1.20) [9]. Importantly, we found in-hospital and 30-day mortality rates of inpatients suffering stage 3 AKI to be 51% and 47%, respectively. In addition, we found that AKI patients were more likely to require ICU admission, mechanical ventilation (invasive mechanical ventilation and non-invasive mechanical ventilation), and had a longer length of hospital stay than patients without AKI, which were also observed in the previous studies [6, 7].

In recent years, lung-kidney crosstalk has become a topic of increasing interest as pertaining to the critically ill patient [20–22]. Firstly, there are several structural similarities and tight functional relationships between lungs and kidneys. Structurally, the alveolar epithelium has similar characteristics to renal tubules in channels localization, cell polarization, and types of water and ion channels [22, 23]. Functionally, both the lungs and kidneys could regulate electrolytes, water, as well as acid–base balances [24]. In the setting of CAP, pulmonary injury may worsen renal function via multiple mechanisms including impact on neurohormonal dysregulation, biotrauma, hemodynamics, remote oxidative stress and cell signaling pathways [22], which may be amplified by mechanical ventilation [25]. Conversely, renal injury may also worsen pulmonary function via multiple mechanisms including uremic toxins, acid imbalance, electrolyte imbalance, inflammation, and oxidative stress [26–29]. Once CAP patients developing with AKI, their prognosis markedly worsens. In the future, more studies should be conducted to further explore lung-kidney crosstalk; such research would be greatly beneficial for improving the prognosis of CAP patients.

There were several limitations in our study. First, it was a single-center retrospective study. In the future, a multicenter prospective study will be conducted to confirm our findings. Second, AKI was defined by SCr and urine output levels according to KDIGO criteria. However, we did not obtain urine output data. Therefore, our analysis did not include the urine output standard of AKI. Third, CURB-65 [13] and Pneumonia Severity Index (PSI) [30] were two scoring systems that estimated the severity of CAP. Ahsan et al. reported that PSI was a significant risk factor for AKI in CAP patients [6]. However, data of PSI was not obtained in this study. Fourth, as this was an observational study, it could only report the associations between AKI and “use of drugs”; yet the temporal relationship between AKI and drug use remained indeterminate due to the inability to regard frequent testing of SCr as sufficient to definitively diagnose AKI. Fifth, as a retrospective study, actual baseline SCr data was lacking. We defined baseline SCr as the lowest level of SCr during hospitalization [31]. As the lowest SCr obtained during a hospitalization is usually equal to or greater than the baseline, it was entirely possible that some patients had already suffered AKI prior to their admission to hospital [31]. Lastly, as it is a retrospective study, some variables, such as most frequent causative pathogens and bacteriemia, were not available.

## Conclusion

AKI was a common complication in Chinese patients with CAP. Patients with CAP who developed AKI had worse in-hospital outcomes.

## Supplementary Information

**Additional file 1.** Factors associated with in-hospital death and 30-days mortality.

## Data Availability

Datasets are available from the corresponding author upon reasonable request.

## Abbreviations

CAP: Community acquired pneumonia
AKI: Acute kidney injury
SCr: Serum creatinine
KDIGO: Kidney Disease: Improving Global Outcomes
COPD: Chronic obstructive pulmonary disease
ARB: Angiotensin receptor blocker
CURB-65: Confusion, urea nitrogen, respiratory rate, blood pressure, and age ≥ 65 years
ICU: Intensive care unit
OR: Odds ratio
HR: Hazard ratio
PSI: Pneumonia Severity Index
ROC: Receiver operating characteristic curve
AUC: Area under curve
