# Supplementary material for: Impact of acute kidney injury on in-hospital outcomes in Chinese patients with community acquired pneumonia
Source: BMC Pulm Med. 2021 May 1;21:143. doi: 10.1186/s12890-021-01511-9 (PMC8088559; doi:10.1186/s12890-021-01511-9)
Supplement: Supplementary file 1 — Additional file 1. Factors associated with in-hospital death and 30-days mortality. [file 12890_2021_1511_MOESM1_ESM.docx]

Table S1 Univariate and multivariable Cox regression analysis determining covariate factors associated with in-hospital death

| Variable | Univariate Cox regression analysis | | | Multivariate Cox regression analysis | | |
| --- | --- | --- | --- | --- | --- | --- |
|  | Survival (n = 3829) | Death (n = 384) | *P* Value | HR | 95% CI | *P* Value |
| **Demographic** |  |  |  |  |  |  |
| Age (years) | 74 (61–83) | 82 (73–87) | < 0.001 |  |  |  |
| Men (%) | 2290 (59.8) | 267 (69.5) | 0.120 |  |  |  |
| **Comorbidity, n (%)** |  |  |  |  |  |  |
| COPD | 423 (11.0) | 56 (14.6) | 0.153 |  |  |  |
| Chronic cor pulmonale | 133 (3.5) | 15 (3.9) | 0.497 |  |  |  |
| Pulmonary arterial hypertension | 121 (3.2) | 7 (1.8) | 0.424 |  |  |  |
| Hypertension | 1861 (48.6) | 230 (59.9) | 0.034 | 1.25 | 1.01–1.55 | 0.049 |
| Atrial fibrillation | 425 (11.1) | 60 (15.6) | 0.970 |  |  |  |
| Cardiac dysfunction | 830 (21.7) | 100 (26.0) | 0.322 |  |  |  |
| Diabetes | 747 (19.5) | 82 (21.4) | 0.813 |  |  |  |
| Chronic kidney disease | 239 (6.2) | 41 (10.7) | 0.045 |  |  |  |
| Cerebrovascular disease | 1121 (29.3) | 212 (55.2) | 0.001 |  |  |  |
| Cancer | 328 (8.6) | 48 (12.5) | 0.307 |  |  |  |
| Rheumatic Diseases | 109 (2.8) | 8 (2.1) | 0.739 |  |  |  |
| **Complication, n (%)** |  |  |  |  |  |  |
| Acute respiratory failure | 487 (12.7) | 218 (56.8) | < 0.001 | 1.98 | 1.52–2.58 | < 0.001 |
| Acute kidney injury | 727 (19.0) | 223 (58.1) | < 0.001 | 1.31 | 1.04–1.66 | 0.023 |
| **Treatment, n (%)** |  |  |  |  |  |  |
| Statin | 714 (18.6) | 63 (16.4) | 0.056 |  |  |  |
| ARB | 548 (14.3) | 37 (9.6) | < 0.001 | 0.47 | 0.33–0.67 | < 0.001 |
| Diuretic | 1383 (36.1) | 288 (75.0) | < 0.001 |  |  |  |
| Vasoactive drugs | 311 (8.1) | 216 (56.3) | < 0.001 | 2.76 | 2.14–3.66 | < 0.001 |
| Mechanical ventilation | 401 (10.5) | 222 (57.8) | < 0.001 |  |  |  |
| ICU admission | 531 (13.9) | 250 (65.1) | < 0.001 |  |  |  |
| **Severity scoring** |  |  |  |  |  |  |
| CURB-65 | 1 (0–2) | 2 (2–3) | < 0.001 | 1.39 | 1.25–1.55 | < 0.001 |

Abbreviation: COPD: chronic obstructive pulmonary disease; ARB: angiotensin receptor blocker; ICU: intensive care unit.

Table S2 Univariate and multivariable Cox regression analysis determining covariate factors associated with 30-days mortality

| Variable | Univariate Cox regression analysis | | | Multivariate Cox regression analysis | | |
| --- | --- | --- | --- | --- | --- | --- |
|  | Survival (n = 3843) | Death (n = 370) | *P* Value | HR | 95% CI | *P* Value |
| **Demographic** |  |  |  |  |  |  |
| Age (years) | 74 (61–83) | 82 (73–87) | < 0.001 |  |  |  |
| Men (%) | 2300 (59.8) | 257 (69.5) | 0.154 |  |  |  |
| **Comorbidity, n (%)** |  |  |  |  |  |  |
| COPD | 425 (11.1) | 54 (14.6) | 0.124 |  |  |  |
| Chronic cor pulmonale | 134 (3.5) | 14 (3.8) | 0.479 |  |  |  |
| Pulmonary arterial hypertension | 121 (3.1) | 7 (1.9) | 0.454 |  |  |  |
| Hypertension | 1868 (48.6) | 223 (60.3) | 0.021 | 1.28 | 1.02–1.59 | 0.030 |
| Atrial fibrillation | 430 (11.2) | 55 (14.9) | 0.885 |  |  |  |
| Cardiac dysfunction | 833 (21.7) | 97 (26.2) | 0.380 |  |  |  |
| Diabetes | 750 (19.5) | 79 (21.4) | 0.814 |  |  |  |
| Chronic kidney disease | 241 (6.3) | 39 (10.5) | 0.064 |  |  |  |
| Cerebrovascular disease | 1130 (29.4) | 203 (54.9) | 0.001 |  |  |  |
| Cancer | 329 (8.6) | 47 (12.7) | 0.253 |  |  |  |
| Rheumatic Diseases | 109 (2.8) | 8 (2.2) | 0.739 |  |  |  |
| **Complication, n (%)** |  |  |  |  |  |  |
| Acute respiratory failure | 496 (12.9) | 209 (56.5) | < 0.001 | 2.01 | 1.54–2.63 | < 0.001 |
| Acute kidney injury | 738 (19.2) | 212 (57.3) | < 0.001 | 1.29 | 1.02–1.62 | 0.033 |
| **Treatment, n (%)** |  |  |  |  |  |  |
| Statin | 714 (18.6) | 63 (17.0) | 0.100 |  |  |  |
| ARB | 552 (14.3) | 33 (8.9) | < 0.001 | 0.47 | 0.33–0.68 | < 0.001 |
| Diuretic | 1392 (36.2) | 279 (75.4) | < 0.001 |  |  |  |
| Vasoactive drugs | 321 (8.4) | 206 (55.7) | < 0.001 | 2.72 | 2.10–3.52 | < 0.001 |
| Mechanical ventilation | 411 (10.7) | 212 (57.3) | < 0.001 |  |  |  |
| ICU admission | 542 (14.1) | 239 (64.6) | < 0.001 |  |  |  |
| **Severity scoring** |  |  |  |  |  |  |
| CURB-65 | 1 (0–2) | 2 (2–3) | < 0.001 | 1.41 | 1.27–1.57 | < 0.001 |

Abbreviation: COPD: chronic obstructive pulmonary disease; ARB: angiotensin receptor blocker; ICU: intensive care unit.
